# Systemic immune-inflammation index and all-cause and cause-specific mortality in sarcopenia: a study from National Health and Nutrition Examination Survey 1999-2018

**DOI:** 10.3389/fimmu.2024.1376544

**Published:** 2024-04-04

**Authors:** Qing-Yue Zeng, Yu Qin, Yi Shi, Xing-Yu Mu, Shi-Jun Huang, Yu-Hao Yang, Si-Min Liu, Zhen-Mei An, Shuang-Qing Li

**Affiliations:** ^1^ General Practice Ward/International Medical Center Ward, General Practice Medical Center, National Clinical Research Center for Geriatrics, West China Hospital, Sichuan University, Chengdu, Sichuan, China; ^2^ Department of Critical Care Medicine, West China Hospital, Sichuan University/West China School of Nursing, Sichuan University, Chengdu, China; ^3^ Department of Endocrinology and Metabolism, West China Hospital, Sichuan University, Chengdu, Sichuan, China

**Keywords:** systemic immune-inflammation index, sarcopenia, mortality, population-based study, NHANES

## Abstract

**Background:**

Sarcopenia, common in the elderly, often linked to chronic diseases, correlates with inflammation.The association between SII and mortality in sarcopenia patients is underexplored, this study investigates this relationship in a U.S. adult cohort.

**Methods:**

We analyzed 1999–2018 NHANES data, focusing on 2,974 adults with sarcopenia. Mortality outcomes were determined by linking to National Death Index (NDI) records up to December 31, 2019. Using a weighted sampling design, participants were grouped into three groups by the Systemic Immune-Inflammation Index (SII). We used Cox regression models, adjusting for demographic and clinical variables, to explore SII’s association with all-cause and cause-specific mortality in sarcopenia, performing sensitivity analyses for robustness.

**Results:**

Over a median follow-up of 9.2 years, 829 deaths occurred. Kaplan-Meier analysis showed significant survival differences across SII groups. The highest SII group showed higher hazard ratios (HRs) for all-cause and cause-specific mortality in both crude and adjusted models. The highest SII group had a higher HR for all-cause(1.57, 1.25–1.98), cardiovascular(1.61, 1.00–2.58), cancer(2.13, 1.32–3.44), and respiratory disease mortality(3.21, 1.66–6.19) in fully adjusted models. Subgroup analyses revealed SII’s association with all-cause mortality across various demographics, including age, gender, and presence of diabetes or cardiovascular disease. Sensitivity analyses, excluding participants with cardiovascular diseases, those who died within two years of follow-up, or those under 45 years of age, largely reflected these results, with the highest SII group consistently demonstrating higher HRs for all types of mortality in both unadjusted and adjusted models.

**Conclusion:**

Our study is the first to demonstrate a significant relationship between SII and increased mortality risks in a sarcopenia population.

## 1 Introduction

Sarcopenia, a skeletal muscle disorder prevalent among the elderly, is characterized by diminished muscle strength, mass, and function. This condition is often aggravated by chronic comorbidities such as cardiovascular diseases, chronic kidney disease, and cancer (1). The prevalence of sarcopenia varies regionally and with age, affecting 1% to 29% of the community-dwelling population and 14% to 33% in long-term care settings (2). Sarcopenia leads to a range of adverse clinical outcomes, including increased risks of recurrent falls and fractures, the development of physical disabilities, more frequent need for hospital services or hospitalization, reduced quality of life, and elevated mortality risk (3). Furthermore, sarcopenia is associated with other conditions, including metabolic and respiratory disorders, and cardiovascular diseases (1, 4–7). The etiology and mechanisms underlying sarcopenia’s pathophysiology are still largely unknown (8). However, current research indicates that inflammation may play a crucial role in the development of sarcopenia, affecting both the functionality and structure of skeletal muscle. Recent meta-analyses demonstrate that individuals with diminished muscle strength and mass have higher levels of inflammatory biomarkers than those in the general population (9, 10).

The Systemic Immune-Inflammation Index (SII), defined as the product of platelet count and the neutrophil-to-lymphocyte ratio, was originally developed to evaluate the prognosis of hepatocellular carcinoma (11). Demonstrating robustness and consistency, SII effectively indicates both local and systemic inflammatory responses in the human body. Previous research has utilized SII to predict and assess the prognosis of various solid tumors, such as gastric cancer (12), non-small cell lung cancer (13), and colorectal cancer (14). Additionally, SII has shown significant prognostic value in cardiovascular and cerebrovascular diseases (15).

Research has shown that inflammation significantly impacts skeletal muscle function and composition, playing a crucial role in the onset of sarcopenia (9, 10, 16). The inflammatory response is intimately linked to sarcopenia’s pathogenesis. Yet, the correlation between the Systemic Immune-Inflammation Index (SII) and long-term clinical outcomes, particularly mortality, in sarcopenia patients remains underexplored. To address this gap, our study investigates the association between SII and both all-cause and cause-specific mortality, considering sarcopenia status, in a nationally representative cohort of United States adults.

## 2 Methods

### 2.1 Study population

The National Health and Nutrition Examination Survey (NHANES) is a national survey that assesses health and health-related behaviors. It employs a complex, clustered, multistage, stratified probability sampling design to achieve a representative sample of the non-institutionalized civilian population of the United States. NHANES integrates interviews and physical examinations to gather demographic, dietary, medical, laboratory, and questionnaire data, as detailed on its website (https://www.cdc.gov/nchs/nhanes/). The National Center for Health Statistics Research Ethics Review Board has approved NHANES data collection, and participants provide written informed consent.

In our study, we analyzed data from eight NHANES cycles spanning 1999–2018: 1999–2000, 2001–2002, 2003–2004, 2005–2006, 2011–2012, 2013–2014, 2015–2016, and 2017–2018. The source for this data is the National Center for Health Statistics, US Centers for Disease Control and Prevention, as detailed in the National Health and Nutrition Examination Survey Documentation Files, available at: https://www.cdc.gov/nchs/nhanes/ (accessed December 15, 2021).

**Figure 1:**
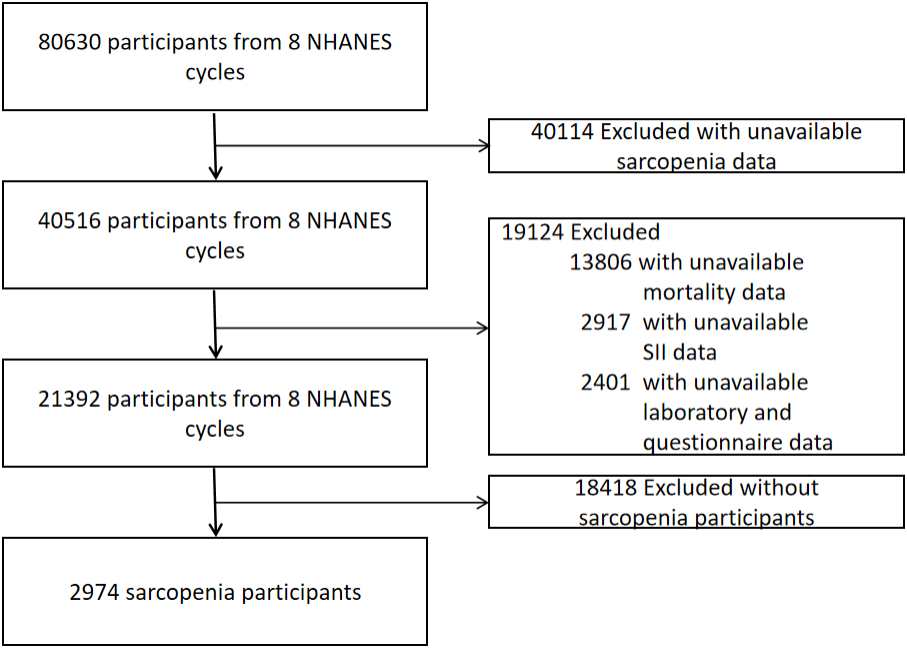
Flowchart of the selection strategy.

The inclusion criteria for our analysis were: (1) participants aged 20 years or older from the 1999–2018 NHANES datasets; (2) availability of data on appendicular skeletal muscle and height; and (3) presence of relevant mortality follow-up and laboratory data for calculating the Systemic Immune-Inflammation Index (SII). The exclusion criteria included: (1) individuals weighing over 136 kg or taller than 192 cm, due to the limitations of the dual-energy X-ray absorptiometry (DXA) scan; (2) pregnant women and individuals with allergies to contrast agents; and (3) those who had been exposed to contrast agents or radioactive therapy within the preceding 7 days. As depicted in **Figure 1**, our final dataset comprised 2,974 participants with sarcopenia, complete data for SII, and mortality information.

### 2.2 Systemic immune-inflammation index and other covariates

The complete blood count is conducted using the Coulter^®^ DxH 800 analyzer by trained personnel. The Systemic Immune-Inflammation Index (SII) is calculated as the product of peripheral platelet and neutrophil counts divided by lymphocyte counts (11). Demographic and laboratory data were collected from the NHANES database for the eight NHANES cycles. The demographic data encompassed age, sex, and race (categorized as Mexican American, non-Hispanic Black, non-Hispanic White, other Hispanic, and Other, including multiracial). Educational attainment was classified into five levels: less than 9th grade, 9-11th grade, high school graduate, some college or associate degree, and college graduate or higher. Family income was categorized into three tiers based on the poverty income ratio: low (≤1.3), medium (>1.3 to ≤3.5), and high (>3.5) (17). Marital status was grouped into married/living with a partner, never married, and other (including widowed, divorced, or separated). Smoking status was divided into never smoked (fewer than 100 cigarettes in lifetime), former smoker (100 cigarettes or more but quit), and current smoker. Alcohol consumption was categorized as never (< 12 lifetime drinks), former (≥ 12 yearly drinks but abstinent last year), current light drinker (≤ 1 daily drink for women, ≤ 2 for men), current moderate drinker (2 daily drinks for women, 3 for men), or current heavy drinker (> 2 daily drinks for women, > 3 for men). Medical conditions included diabetes mellitus and cardiovascular disease (CVD), identified either through self-reports or laboratory/imaging results. Diabetes mellitus(DM) diagnosis criteria included a self-reported diagnosis, use of anti-diabetic drugs, fasting glucose levels ≥7.0 mmol/L (126 mg/dL), random or two-hour oral glucose tolerance test glucose ≥11.1 mmol/L, or an HbA1c level ≥ 6.5% (18). CVD was determined using standardized medical questionnaires and self-reported physician diagnoses, with any positive response to diagnoses of coronary heart disease, congestive heart failure, heart attack, angina, or stroke indicating CVD. Laboratory data included aspartate aminotransferase (AST),alanine aminotransferase (ALT), triglycerides(TG),total cholesterol, high density lipoprotein cholesterol(HDL-cholesterol),glycosylated hemoglobin (HbA1c), urinary albumin creatinine ratio(UACR), white blood cells(WBC), platelet (PLT), lymphocyte (Lym) and neutrophil (Neu).

### 2.3 Definition of sarcopenia

Appendicular Skeletal Mass (ASM) is frequently used to represent skeletal muscle mass. Within the commonly accepted definitions of sarcopenia, the Appendicular Skeletal Mass Index (ASMI), derived from ASM adjusted for height, is the most prevalent metric (19).

ASM is determined as the total muscle mass of the arms and legs, measured using a DXA QDR-4500 Hologic scanner. ASM, typically adjusted for height, aids in establishing cutoff values for the ASMI. ASMI is calculated using the formula ASMI = ASM/height^2, where ASM is in kilograms (kg) and height is in meters (m). According to the European Working Group on Sarcopenia in Older People (EWGSOP) criteria, sarcopenia is diagnosed based on ASMI values, defined as ≤7.26 kg/m^2 for men and ≤5.5 kg/m^2 for women (19). To ensure accuracy, participants removed metal objects, in addition to false teeth and hearing aids, prior to the measurement. ASM was assessed for each participant, and each NHANES cycle followed this consistent procedure.

### 2.4 All-cause and cause-specific mortality

To determine the mortality status of the follow-up population, we used the NHANES public-use linked mortality file updated as of December 31, 2019. Follow-up duration was determined in months, starting from the participant’s visit to the mobile examination center to either the date of death or the end of the mortality follow-up period. Cause-specific mortality was classified according to the International Classification of Diseases, Tenth Revision (ICD-10). The primary outcomes included all-cause mortality and three specific causes of death: cardiovascular mortality (coded I00-I09, I11, I13, I20-I51, and I60-I69), cancer mortality (coded C00-C97), and respiratory diseases mortality (coded J09-J18, J40-J47).

### 2.5 Statistical analysis

In alignment with NHANES analytic guidelines, our analyses factored in the complex sampling design and sampling weights. Since there is currently no normal cutoff value for SII, we divided all participants into three groups based on the tertiles of the Systemic Immune-Inflammation Index (SII). Participant characteristics were presented as weighted means (Standard Error) for continuous variables and as numbers and weighted proportions for categorical variables. For continuous data, weighted linear regression was employed, while categorical data was analyzed using the design-adjusted chi-square test.

To investigate the independent association of the SII with all-cause and cause-specific mortality in sarcopenia, we utilized Cox proportional hazards regression analysis, controlling for potential demographic and clinical confounders. Four models were developed: Crude model; Models 1, 2, and 3 were used as adjusted models. Model 1 included gender and age; Model 2 added race, education level, family poverty income ratio, and marital status to the parameters of Model 1; Model 3 further included BMI, ALT, AST, TG, total cholesterol, HDL-cholesterol, HbA1c, UACR, DM, and drinking and smoking status. The graphical association between log Hazard Ratio (log HR) for SII parameter and mortality was assessed using restricted cubic spline (RCS) plots with three knots. For the assessment of all-cause mortality, interaction and subgroup analyses were conducted, taking into account various factors such as age group (with a threshold at age 60), sex, presence of DM, CVD, ethnicity, marital status, and categorizations of C-reactive protein (CRP) and total cholesterol into three distinct groups each. These analyses utilized a Multivariable Cox Proportional Hazards Model, specifically based on Model 3. Likewise, for cause-specific mortality, analyses were conducted based on factors such as age, sex, DM, CVD, and levels of CRP and total cholesterol, utilizing the identical model framework. To reinforce the reliability of our results, we conducted three sensitivity analyses. Firstly, we excluded participants who passed away within two years of follow-up, aiming to eliminate potential reverse causality effects. Secondly, acknowledging the possible impact of pre-existing CVD on mortality outcomes, we carried out an additional sensitivity analysis, specifically excluding individuals with a history of CVD.Thirdly, we excluded participants under the age of 45 to assess this relationship. Furthermore, to investigate whether the SII has a stronger impact on mortality prognosis in patients with sarcopenia compared to those without, we divided the SII into two categories based on the median value, resulting in four groups: low SII without sarcopenia (low SII/SP-), low SII with sarcopenia (low SII/SP+), high SII without sarcopenia (high SII/SP-), and high SII with sarcopenia (high SII/SP+).Statistical significance was set at p < 0.05. All analyses were performed using R software version 4.3.1 (R Foundation, Vienna, Austria).

## 3 Results

### 3.1 Baseline characteristics of the study population

Upon screening the eight cycles of NHANES data, 2,974 individuals met our inclusion criteria and were subsequently included in our final analysis. The selection process is detailed in **Figure 1**. Demographic and laboratory data of the 2,974 enrolled participants are summarized in **Table 1**. The study population was segmented into three groups according to tertiles of the SII, with the cutoff values for SII established at 402.8 and 626.5: the first tertile group (Q1, n = 992; SII ≤ 402.8), the second tertile group (Q2, n = 990; 402.8 < SII ≤ 626.5), and the third tertile group (Q3, n = 992; SII > 626.5). The mean age of the entire cohort was 45.08 years, with males constituting 46.75%. The average SII was 588.86 (Standard Error 9.96). Participants in the Q3 group, compared to those in Q1, tended to be older, less likely male, predominantly Non-Hispanic White, mostly married, and heavy drinkers. Additionally, the prevalence of DM and CVD increased with higher SII levels, as detailed in **Table 1**.

**Table 1:** Characteristics of sarcopenia participants in the NHANES.

Variables	Total (n=2974)	Q1 (23.8,402.8) (n=992)	Q2 (402.8,626.5) (n=990)	Q3(626.5,4972.5) (n-992)	Pvalue
Age years mean (SD)	45.08(0.41)	42.15(0.70)	45.13(0.63)	48.13(0.72)	<0.0001
BMI, kg/m2 mean (SD)	21.58(0.06)	21.22(0.10)	21.82(0.11)	21.73(0.10)	<0.0001
Sex, (%)					0.06
Female	1474(53.25)	466(49.67)	500(53.96)	508(56.31)	
Male	1500(46.75)	526(50.33)	490(46.04)	484(43.69)	
Ethnicity, n (%)					<0.0001
Mexican American	538(6.75)	153(6.19)	196(7.18)	189(6.91)	
Non-Hispanic Black	214(3.64)	114(6.09)	60(2.72)	4012.00)	
Non-Hispanic White	1591(74.46)	465(69.91)	528(75.90)	598(77.77)	
Other Hispanic	151(4.41)	53(4.49)	43(3.48)	55(5.28)	
Other Race Including Multi-Racial	480(10.74)	207(13.32)	163(10.72)	110(8.04)	
Educational level, n. (%)					0.24
9-11th Grade (Includes 12th grade with no diploma)	414(11.40)	141(11.61)	142(11.64)	131(10.92)	
College Graduate or above	724(29.00)	258(30.79)	234(28.61)	232(27.52)	
High School Grad/GED or Equivalent	685(24.87)	219(23.13)	225(25.09)	241(26.48)	
Less Than 9th Grade	388(5.92)	107(4.11)	137(6.07)	144(7.68)	
Some College or AA degree	763(28.81)	267(30.37)	252(28.59)	244(27.40)	
Family income, n (%)					0.35
High	910(39.17)	317(40.67)	316(40.06)	277(36.67)	
Low	936(23.27)	315(21.36)	314(24.66)	307(23.86)	
Medium	1128(37.57)	360|37.97)	360(35.28)	408(39.47)	
Marital status, n (%)					<0.0001
Living with partner	194(7.48)	92(10.75)	51(5.80)	51[5.74)	
Married	1518(51.23)	476|48.60)	530(53.61)	512(51.56)	
Never married	634(23.91)	242(26.88)	213(24.04)	179(20.65)	
other	628(17.39)	182(13.76)	196(16.54)	250(22.06)	
Smoking status, n (%)					0.09
former	682(21.61)	202(21.64)	224(20.37)	256(22.84)	
never	1489(47.21)	524148.421	519(50.51)	446(42.57)	
now	803(31.19)	266129.94	247(29.12)	290(34.60)	
Alcohol user, n (%)					0.02
former	525(13.55)	144|10.84	164(12.88)	217(17.08)	
heavy	581(22.11)	190(21.71)	187(21.36)	204(23.28)	
mild	977(34.13)	343(37.69)	348(35.90)	286(28.58)	
moderate	400(17.07)	135(15.83)	129(16.75)	136(18.71)	
never	491(13.14)	180(13.93)	162(13.11)	149(12.36)	
DM, (%)					0.43
yes	286(6.21)	85(5.74)	91(5.68)	110(7.23)	
no	2688(93.79)	907(94.26)	899(94.32)	882(92.77)	
CVD, (%)					<0.001
yes	311(6.85)	79(4.78)	91(6.24)	141(9.65)	
no	2663(93.15)	913(95.22)	899(93.76)	851(90.35)	
ALT, U/L, mean (SD)	21.93(0.38)	22.64(0.69)	22.33(0.82)	20.78(0.39)	0.03
AST, U/L	24.42(0.38)	25.53(0.79)	24.37(0.67)	23.30(0.46)	0.06
Triglycerides, mmol/L mean (SD)	1.33(0.03)	1.25(0.04)	1.34(0.03)	1.40(0.08)	0.06
Total cholesterol, mmol/L median[Q1, Q3)	4.97(4.29,5.64)	4.81(4.19,5.53)	5.04(4.34,5.66)	5.02(4.40,5.72)	0.002
HDL-cholesterol, mmol/L mean (SD)	1.53(0.01)	1.53(0.02)	1.53(0.02)	1.53(0.02)	0.98
HbA1c, % mean (SD)	5.3610.02)	5.33(0.03)	5.37(0.03)	5.37(0.02)	0.59
UACR, mg/e. medianjQ1 Q3)	7.31(4.74,13.04)	6.75(4.44,11.01)	7.20(4.74,13.02)	8.18(5.08,15.71)	<0.0001
SII. (1,000 cells/ul) mean (SD)	588.86(9.96)	302.51(3.45)	508.22(2.40)	972.34(17.57)	<0.0001

### 3.2 SII and all-cause and cause-specific mortality

**Figure 2:**
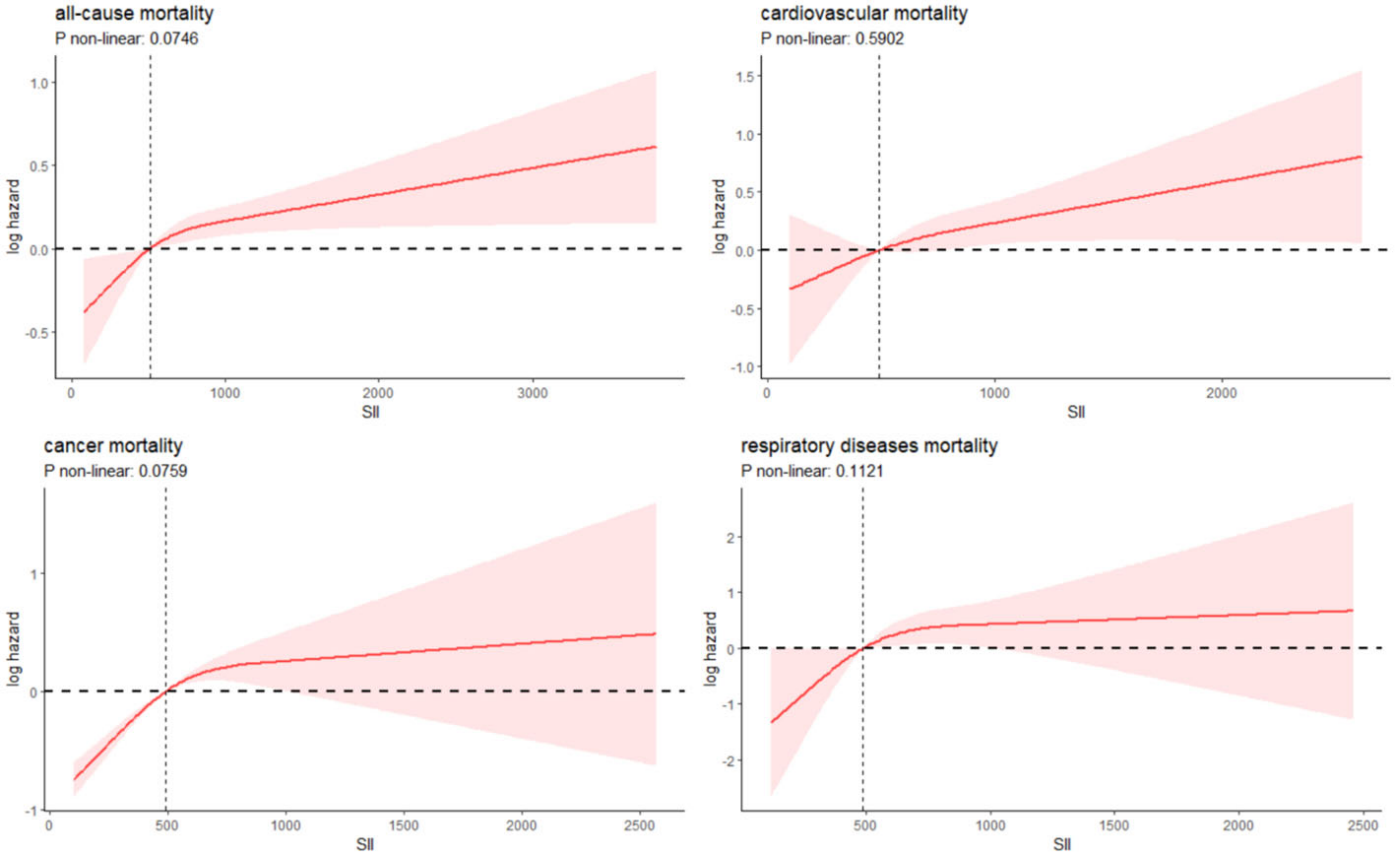
Restricted cubic spline fitting for the association between SII with mortality.

**Figure 3:**
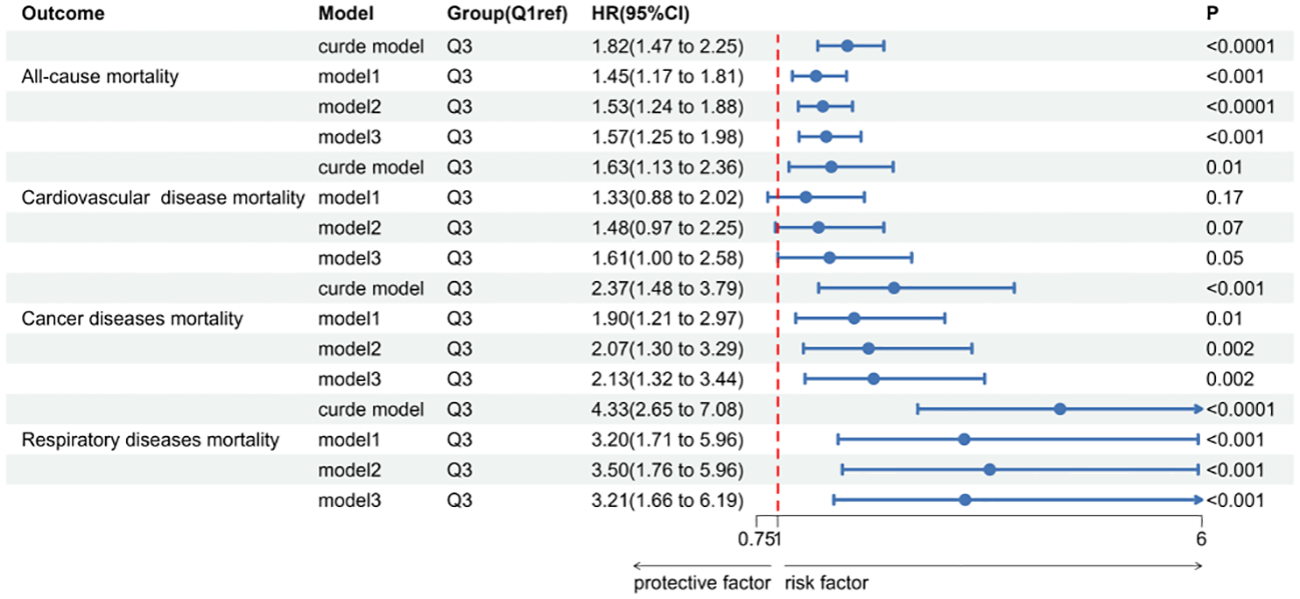
The forest map of Systemic immune-Inflammation Index(SII) in sarcopenia and all-cause and cause-specific mortality.

**Figure 4:**
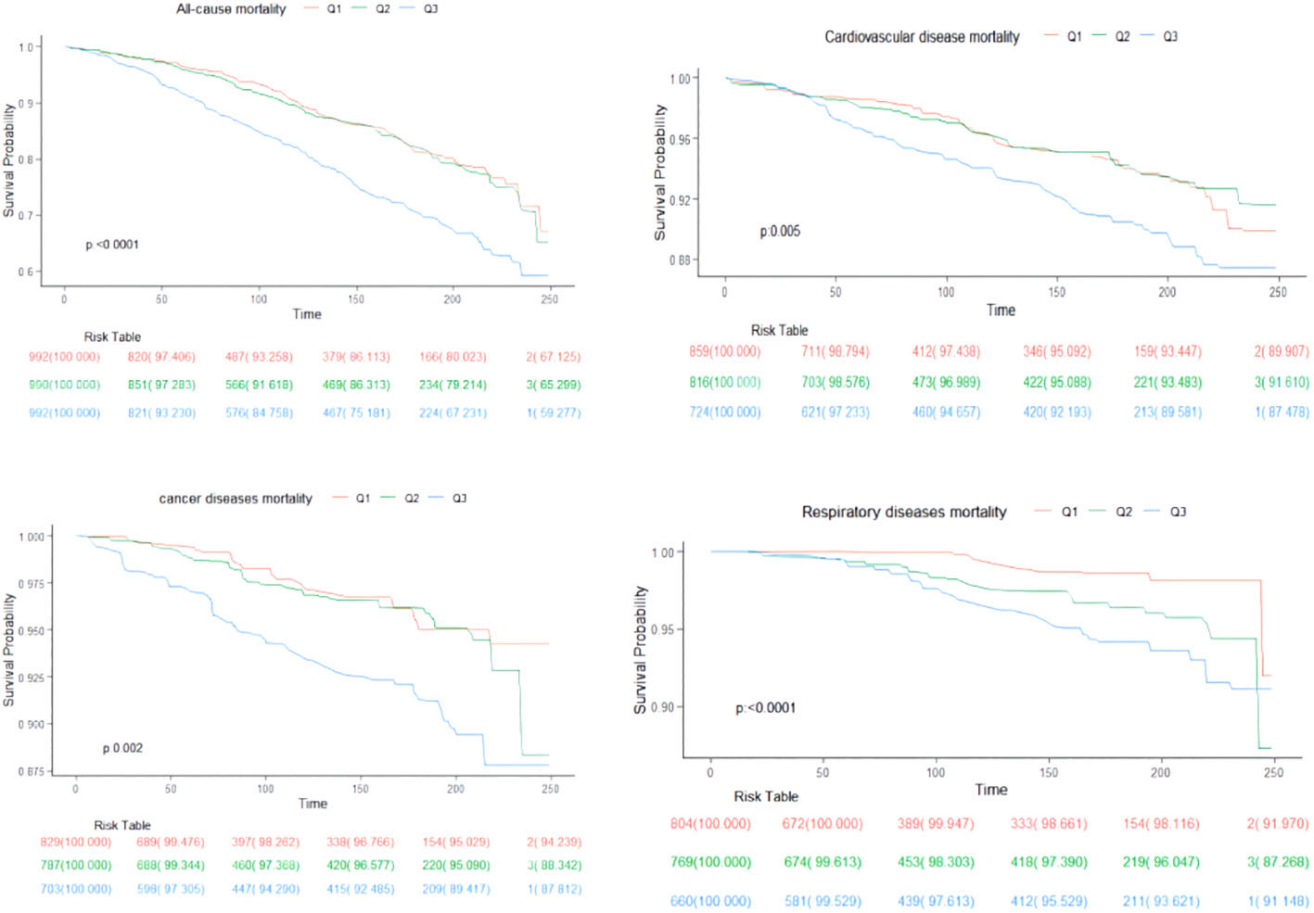
Kaplan-Meier curves show all-cause and cause-specific mortality differences by SII.

Over a median follow-up period of 9.2 years (interquartile range: 5.3, 16), there were 829 deaths in total. Breaking down cause-specific mortality, there were 254 cardiovascular-related deaths, 174 cancer-related deaths, and 88 deaths due to chronic lower respiratory diseases. The RCS plot analysis indicated a continuous association between higher SII and an increased risk of all-cause mortality and cause-specific mortality (**Figure 2**). Survival analysis results are presented in **Supplementary Table S1**; **Figure 3**. Kaplan-Meier analysis indicated statistically significant differences in survival probabilities among the three SII groups (p-values ranging from 0.005 to <0.0001, as shown in **Figure 4**). In the crude model, the hazard ratio (HR) for the third tertile group (Q3) was 1.82 (95% CI, 1.47–2.25) for all-cause mortality, 1.63 (95% CI, 1.13–2.36) for cardiovascular mortality, 2.37 (95% CI, 1.48–3.79) for cancer mortality, and 4.33 (95% CI, 2.65–7.08) for respiratory disease mortality. In the fully adjusted model (Model 3), the HRs for Q3 were 1.57 (95% CI, 1.25–1.98) for all-cause mortality, 1.61 (95% CI, 1.00–2.58) for cardiovascular mortality, 2.13 (95% CI, 1.32–3.44) for cancer mortality, and 3.21 (95% CI, 1.66–6.19) for respiratory disease mortality. Additionally, the p-value for the trend of SII levels was calculated in each model to determine the presence of a nonlinear relationship between SII and mortality outcomes, as detailed in **Supplementary Table S1** (p for trend 0.04 to <0.0001).

### 3.3 Subgroup analyses and sensitivity analyses

The subgroup analyses for all-cause mortality and cause-specific mortality, presented in **Table 2**, reveal that the SII is significantly associated with all-cause mortality across various subgroups. Specifically, the associations were observed in participants aged 20 to 60 years (HR, 1.87; 95% CI, 1.21-2.90), those aged 61 to 85 years (HR, 1.59; 95% CI, 1.30-1.95), males (HR, 2.18; 95% CI, 1.64-2.90), females (HR, 1.56; 95% CI, 1.10-2.31), individuals with DM (HR, 2.71; 95% CI, 1.62-4.53), those without DM (HR, 1.69; 95% CI, 1.32-2.15), with CVD (HR, 1.63; 95% CI, 1.10-2.43), and without CVD (HR, 1.73; 95% CI, 1.33-2.25), Non-Hispanic Whites (HR, 1.90; 95% CI, 1.50-2.41), married individuals (HR, 1.97; 95% CI, 1.43-2.70), those with other marital statuses (HR, 1.65; 95% CI, 1.23-2.20), higher levels of CRP (HR, 1.67; 95% CI, 1.24-2.26), moderate total cholesterol (HR, 1.85; 95% CI, 1.22-2.79), and higher total cholesterol (HR, 2.03; 95% CI, 1.49-2.78). However, no significant association was found for moderate and low levels of CRP, low total cholesterol, and the remaining ethnic and marital status groups. The results of the subgroup analyses for cause-specific mortality are thoroughly detailed in **Supplementary Tables S2A-S2C**.

**Table 2:** Subgroup of association between systemic immune-Inflammation index (SII) and all-cause mortality.

Subgroup	Q1	Q2 Adjusted HR (95% CI)	P	Q3 Adjusted HR (95% CI)	P	p for trend	p for interaction
AGE							0.732
20-60	ref	1.089(0.636,1.866)	0.756	1.878(1.213,2.908)	0.005	0.004	
60-85	ref	0.952(0.756,1.199)	0.675	1.587(1.291,1.952)	<0.0001	<0.0001	
DM							0.365
no	ref	0.954(0.708,1.288)	0.76	1.686(1.322,2.149)	<0.0001	<0.0001	
yes	ref	1.755(0.983,3.135)	0.057	2.707(1.617,4.532)	<0.001	<0.001	
CVD							0.977
no	ref	0.977(0.706,1.352)	0.886	1.727(1.327,2.247)	<0.0001	<0.0001	
yes	ref	1.059(0.662,1.693)	0.812	1.630(1.094,2.430)	0.016	0.005	
sex							0.058
Female	ref	0.739(0.506,1.081)	0.119	1.562(1.056,2.309)	0.025	0.011	
Male	ref	1.419(1.001,2.012)	0.05	2.179(1.639,2.895)	<0.0001	<0.0001	
CRP							0.288
Q1	ref	1.202(0.601,2.403)	0.602	1.706(0.938,3.101)	0.08	0.086	
Q2	ref	0.864(0.564,1.324)	0.502	1.217(0.835,1.773)	0.306	0.271	
Q3	ref	0.861(0.581,1.275)	0.454	1.673(1.237,2.264)	<0.001	<0.0001	
total cholesterol							0.335
Q1	ref	1.100(0.651,1.860)	0.721	1.678(0.970,2.901)	0.064	0.064	
Q2	ref	1.097(0.688,1.750)	0.696	1.845(1.221,2.789)	0.004	0.003	
Q3	ref	0.935(0.632,1.385)	0.738	2.034(1.487,2.781)	<0.0001	<0.0001	
Ethnicity							0.468
Non-Hispanic White	ref	1.032(0.739,1.442)	0.852	1.900(1.497,2.412)	<0.0001	<0.0001	
Mexican American	ref	0.705(0.374,1.328)	0.28	1.258(0.648,2.440)	0.497	0.428	
Other Race Including	ref	0.758(0.152,3.779)	0.735	2.296(0.559,9.426)	0.249	0.326	
Multi-Racial							
Other Hispanic	ref	0.456(0.085,2.454)	0.36	0.428(0.115,1.591)	0.205	0.205	
Non-Hispanic Black	ref	1.782(0.915,3.472)	0.089	1.803(0.898,3.619)	0.097	0.075	
marital							0.272
other	ref	0.870(0.613,1.235)	0.436	1.645(1.232,2.197)	<0.001	<0.001	
Married	ref	0.901(0.609,1.333)	0.603	1.969(1.434,2.704)	<0.0001	<0.0001	
Living with partner	ref	0.419(0.079,2.224)	0.307	0.211(0.018,2.537)	0.22	0.22	
Never married	ref	1.629(0.665,3.992)	0.286	1.318(0.496,3.499)	0.579	0.579	

The **Supplementary Tables S3A-B, S4A-B, S5A-B**, along with **Supplementary Figures S1–S3**, display baseline data, outcomes from various models, and the results of Kaplan-Meier sensitivity tests. After excluding participants with CVD, the crude model showed that for the third tertile group (Q3), the hazard ratio (HR) was 1.70 (95% CI, 1.30–2.22) for all-cause mortality, 1.68 (95% CI, 1.09–2.60) for cardiovascular mortality, 1.82 (95% CI, 1.10–3.01) for cancer mortality, and 4.50 (95% CI, 1.82–11.15) for respiratory disease mortality. In the fully adjusted model (Model 3), the HR for Q3 was 1.55 (95% CI, 1.15–2.09) for all-cause mortality, 1.74 (95% CI, 1.02–2.95) for cardiovascular mortality, 1.75 (95% CI, 1.00–3.07) for cancer mortality, and 3.87 (95% CI, 1.19–12.60) for respiratory disease mortality. In sensitivity analyses, excluding participants who died within two years of follow-up, showed in Model 3 that the HRs for Q3 were 1.62 (95% CI, 1.27–2.08) for all-cause mortality, 1.91 (95% CI, 1.13–3.23) for cardiovascular mortality, 1.91 (95% CI, 1.20–3.03) for cancer mortality, and 3.10 (95% CI, 1.62–5.93) for respiratory disease mortality. Similarly, analyses excluding participants under 45 years old indicated in Model 3 that the HRs for Q3 were 1.67 (95% CI, 1.32–2.11) for all-cause mortality, 1.62 (95% CI, 0.99–2.65) for cardiovascular mortality, 2.24 (95% CI, 1.34–3.74) for cancer mortality, and 3.56 (95% CI, 1.79–7.05) for respiratory disease mortality. Statistical differences were observed in survival probabilities among different tertiles for various disease mortalities across several sensitivity analyses. (**Supplementary Figures S1A-D, S2A-D, S3A-D**).

### 3.4 All-cause and cause-specific mortality according to SII and sarcopenia status

**Table 3** presents the hazard ratios (HRs) for all-cause and cause-specific mortality according to SII and sarcopenia (SP) status. In the fully adjusted model, compared to the low SII/SP- baseline group, an increased risk of all-cause mortality was observed in both the low SII/SP+ group (HR = 1.25, 95% CI: 1.04–1.49, p = 0.02) and the high SII/SP+ group (HR = 1.72, 95% CI: 1.44–2.05, p < 0.0001). However, the high SII/SP- group did not show statistical significance (HR = 1.03, 95% CI: 0.93–1.14, p = 0.55). For cardiovascular mortality, increased risks were also observed in the low SII/SP+ (HR = 1.44, 95% CI: 1.01–2.05, p =0.04) and high SII/SP+ (HR = 1.97, 95% CI: 1.45–2.67, p < 0.0001) groups compared to the low SII/SP- group, while the high SII/SP- group again did not reach statistical significance (HR = 1.17, 95% CI: 0.96–1.42, p = 0.13). Similarly, for heart mortality, elevated risks were noted in the high SII/SP+ group (HR = 1.66, 95% CI: 1.27–2.18, p < 0.001), but not in the low SII/SP+ (HR = 1.08, 95% CI: 0.76–1.53, p = 0.67) and high SII/SP- groups (HR = 0.85, 95% CI: 0.66–1.08, p = 0.18). For respiratory mortality, higher risks were found in the high SII/SP- (HR = 1.69, 95% CI: 1.16–2.47, p =0.01) and high SII/SP+ groups (HR = 4.42, 95% CI: 2.74–7.13, p < 0.0001), while the low SII/SP+ group did not show statistical significance (HR = 1.69, 95% CI: 0.94–3.04, p = 0.08).

Table 3All-cause and cause-specific mortality according to SII and sarcopenia status.

**Table:** 

	All-cause mortality							
	crude model		Model 1		Model 2		Model 3	
group	95%CI	P	95%CI	P	95%CI	P	95%CI	P
low SII/SP-	ref		ref		ref		ref	
low_SII/SP+	1.65(1.38,1.96)	<0.0001	1.18(1.01,1.37)	0.04	1.18(1.00,1.40)	0.05	1.25(1.04,1.49)	0.02
high _SII/SP-	1.00(0.90,1.11)	0.96	1.02(0.93,1.12)	0.67	1.06(0.97,1.16)	0.2	1.03(0.93,1.14)	0.55
high_SII/SP+	2.68(2.33,3.10)	<0.0001	1.61(1.41,1.85)	<0.0001	1.63(1.42,1.87)	<0.0001	1.72(1.44,2.05)	<0.0001
pfor trend		<0.0001		<0.0001		<0.0001		<0.0001

**Table:** 

	Cardiavascular disease mortality							
	crude model		Model 1		Model 2		Model 3	
group	95%CI	P	95%CI	P	95%CI	P	95%CI	P
low_SII/SP-	ref		ref		ref		ref	
low_SII/SP+	1.79(1.32,2.43)	<0.001	1.21(0.88,1.67)	0.24	1.25(0.89,1.75)	0.2	1.44(1.01,2.05)	0.04
high_SII/SP-	1.06(0.86,1.32)	0.58	1.14(0.93,1.40)	0.21	1.24(1.02,1.50)	0,03	1.17(0.96,1.42)	0.13
high_SII/SP+	2.65(2.11,3.32)	<0.0001	1.55(1.24,1.94)	<0.001	1.65(1.30,2.10)	<0.0001	1.97(1.45,2.67)	<0.0001
pfor trend		<0.001		0.004		<0.001		<0.001

**Table:** 

	Cancer Diseases mortality							
	crude model		Model 1		Model 2		Model 3	
group	95%CI	P	95%CI	P	95%CI	P	95%CI	P
low_SII/SP	ref		ref		ref		ref	
low_SII/SP+	1.24(0.89,1.72)	0.21	1.04(0.75,1.43)	0.82	1.03(0.74,1.44)	0.85	1.08(0.76,1.53)	0.67
high SII/SP-	0.81(0.64,1.02)	0.07	0.86(0.69,1.07)	0.18	0.89(0.71,1.11)	0.29	0.85(0.66,1.08)	0.18
high SII/SP+	2.29(1.78,2.96)	<0.0001	1.61(1.25,2.08)	<0.001	1.65(1.26,2.17)	<0.001	1.66(1.27,2.18)	<0.001
p for trend		0.07		0.18		0.13		0.34

**Table:** 

	Respiratory diseases mortality							
	crude model		Model 1		Model 2		Model 3	
group	95%CI	P	95%CI	P	95%CI	P	95%CI	P
low_SII/SP-	ref		ref		ref		ref	
low_SII/SP+	2.48(1.56,3.94)	<0.001	1.75(1.07,2.88)	0.03	1.79(1.07,2.99)	0.03	1.69(0.94,3.04)	0.08
high_SII/SP-	1.51(1.05,2.16)	0.03	1.63(1.13,2.34)	0.01	1.74(1.16,2.62)	0.01	1.69(1.16,2.47)	0.01
high_SII/SP+	8.77(5.76,13.35)	<0.0001	4.60(2.86,7.40)	<0.0001	4.92(3.05,7.95)	<0.0001	4.42(2.74,7.13)	<0.0001
p for trend		<0.0001		<0.0001		<0.0001		<0.0001

## 4 Discussion

Using a representative sample of US adults with sarcopenia, our findings reveal that the Systemic Immune-Inflammation Index (SII) is significantly associated with increased mortality due to all causes, cardiovascular disease, cancer, and respiratory issues in univariate analyses. This association remained significant even after adjusting for full covariates, suggesting a linear correlation between SII levels and mortality risks, where higher SII levels are linked to increased risks. In our subgroup analyses, an increased correlation between elevated SII and higher mortality risk was observed in the majority of subgroups, and this pattern was largely mirrored in the sensitivity analyses, with the third quartile (Q3) consistently showing higher HRs for all types of mortality in both unadjusted and adjusted models. To our knowledge, this is the first study to investigate the relationship between SII and the risk of all-cause and cause-specific mortality within a sarcopenia population. Furthermore, in exploring the prognostic impact of SII on mortality outcomes among patients with and without sarcopenia, we found that SII has a stronger prognostic impact in patients with sarcopenia.

In our subgroup analyses, we observed a consistent correlation between elevated SII levels and increased mortality risk across all subgroups, regardless of gender, age brackets, and the presence or absence of DM and CVD. Interestingly, this correlation was not found in groups with low and moderate levels of CRP and low total cholesterol levels. This absence of correlation might be due to low to moderate CRP levels reflecting mild to moderate inflammatory states, where the impact of inflammation on an individual’s health status may be limited, especially in the short term. Thus, compared to high CRP levels, low to moderate CRP levels may not significantly affect the mortality prognosis in patients with sarcopenia; low total cholesterol levels might be associated with better nutritional status or a lower risk of cardiovascular diseases, which could offset the risks posed by elevated SII levels. In our primary analysis, SII was marginally significant in predicting mortality from cardiovascular disease. Meanwhile, SII was a significant predictor of mortality from cancer and respiratory diseases. Taken together with the results of the subgroup analyses, it is possible that the levels of CRP and total cholesterol influence the correlation between SII and the risk of mortality from cardiovascular disease, as supported by numerous studies demonstrating the correlation between CRP, cholesterol, and the risk of cardiovascular diseases and mortality (20, 21).

An increasing body of research underscores the role of inflammation as a key regulator in the processes governing skeletal muscle homeostasis, ultimately contributing to the development of sarcopenia and its severe complications (22–24). Numerous clinical and epidemiological studies have shown that various plasma inflammatory markers, including CRP, high-sensitivity C-reactive protein, and interleukin-6, are often elevated in patients with sarcopenia and correlate with their prognosis (25–27). A meta-analysis of 17 studies, encompassing 11,249 participants, found that individuals with sarcopenia had significantly higher levels of CRP compared to controls (28). Furthermore, some clinical trials focusing on anti-inflammatory treatments for sarcopenia have reported improvements in muscle strength and function (29, 30). These findings collectively suggest a strong link between inflammation and sarcopenia.

Our findings demonstrate a significant association between the Systemic Immune-Inflammation Index (SII) and increased mortality from all causes, cardiovascular diseases, cancer, and respiratory conditions in patients with sarcopenia. Previous research has established a link between sarcopenia and increased risks of cardiovascular diseases, cancer, and respiratory disorders (15, 31–33). For example, a study from the China Health and Retirement Longitudinal Study, involving 15,137 participants over 3.6 years of follow-up, found that individuals diagnosed with sarcopenia were more likely to develop new onset CVD than their non-sarcopenic counterparts (HR: 1.33, 95% CI: 1.04-1.71) (15). An umbrella review including 30 meta-analyses of sarcopenia and adverse outcomes highlighted significant associations of sarcopenia with poorer prognosis across 12 cancer types including gastric, hepatocellular, urothelial, head and neck, hematologic malignancies, pancreatic, breast, colorectal, lung, esophageal, and ovarian cancers (34). A cross-sectional case-control study of 622 COPD patients revealed that those with sarcopenia had reduced exercise capacity, functional performance, physical activity, and health status compared to patients without sarcopenia (33). A meta-analysis of 56 articles showed that sarcopenia was associated with a significantly higher risk of all-cause mortality (HR: 2.00 [95% CI: 1.71, 2.34]), independent of the population studied, the definition of sarcopenia used, and the follow-up period in subgroup analyses (35). There is substantial evidence linking sarcopenia with increased mortality from cardiovascular disease and cancer (36–38). To our knowledge, this study is the first to prospectively demonstrate a relationship between SII and respiratory-related mortality specifically within the sarcopenia context. The structure of the SII formula suggests that the increased mortality risk associated with SII might be due to complex interactions involving Neutrophil extracellular traps (NETs) in neutrophils, factors like innate immunity, oxidative stress, and cardiovascular diseases (39, 40). Additionally, platelets may increase thrombosis risk and promote tumor development and metastasis through interactions with cancer cells (41, 42). Moreover, lymphocyte-mediated inflammatory responses could cause endothelial dysfunction and organ damage (43).

Current research on the prognosis of patients with sarcopenia frequently emphasizes the relevance of nutrition. In a clinical study spanning 12 weeks, it was found that an Internet-based nutrition intervention enhanced the intake of high-quality protein and increased skeletal muscle mass in elderly individuals with sarcopenia (44). Additionally, a narrative review on sarcopenia focusing on Nutritional and Nutrition-Related Biomarkers identified creatinine as a reliable biomarker for muscle mass status, attributed to its ease of access and cost-effectiveness. Vitamin D status is also recognized as a valuable biomarker for predicting overall mortality (45). The importance of nutrition in the prognosis of sarcopenia is well acknowledged, yet there is a noticeable lack of literature on this subject, highlighting the need for further studies to explore this relationship.

This study has numerous strengths and some limitations. Among its strengths are the high-quality data, meticulously collected by trained professionals adhering to a well-designed protocol, the comprehensive clinical variables available, the large and representative sample of the US population, and the extended follow-up period with a median of 9.3 years for mortality assessment. Sensitivity and subgroup analyses have further reinforced the robustness of our findings. Our results are likely applicable to Western populations with similar social and health behavior patterns. Additionally, our study offers a comprehensive assessment of patient mortality prognosis. However, the study is not without limitations. Firstly, the NHANES dataset lacks follow-up data on SII and muscle-related factors, preventing the assessment of longitudinal changes in SII and sarcopenia. Secondly, due to insufficient data on muscle strength and function in NHANES, these factors were not incorporated into our analysis. Future studies should adopt longitudinal designs to include such variables, potentially offering deeper insights into the relationship between inflammation and muscle metabolism. Thirdly, despite adjusting for multiple confounding factors, the possibility of unaccounted confounders in our analysis and subgroup analysis cannot be entirely excluded.

## 5 Conclusion

In this nationally representative study of US adults, the SII within the context of sarcopenia is linked with increased mortality due to all causes, as well as cardiovascular, cancer, and respiratory diseases, independent of metabolic and demographic risk factors. These findings underscore the independent prognostic significance of SII in patients with sarcopenia. Given the notable correlation between SII and mortality across various categories in individuals with sarcopenia, exploring interventions aimed at reducing inflammation could be beneficial. These might include pharmaceutical approaches, physical activity, and dietary modifications, all of which could potentially enhance the prognosis for sarcopenia patients.

## Data availability statement

The original contributions presented in the study are included in the article/**Supplementary Material**. Further inquiries can be directed to the corresponding authors.

## Ethics statement

The requirement of ethical approval was waived by The National Center for Health Statistics Research Ethics Review Board for the studies on humans because The National Center for Health Statistics Research Ethics Review Board has approved NHANES data collection, and participants provide written informed consent. The studies were conducted in accordance with the local legislation and institutional requirements. Written informed consent for participation was not required from the participants or the participants’ legal guardians/next of kin in accordance with the national legislation and institutional requirements. The human samples used in this study were acquired from gifted from another research group.

## Author contributions

Q-YZ: Validation, Supervision, Formal analysis, Data curation, Writing – review & editing, Writing – original draft. YQ: Writing – review & editing, Formal analysis. YS: Writing – review & editing, Software, Conceptualization. X-YM: Writing – review & editing, Validation. S-JH: Writing – review & editing, Validation. Y-HY: Writing – review & editing, Data curation. S-ML: Writing – review & editing, Data curation. Z-MA: Writing – review & editing. S-QL: Writing – review & editing, Funding acquisition.
