# Supplementary material for: Systemic immune-inflammation index and all-cause and cause-specific mortality in sarcopenia: a study from National Health and Nutrition Examination Survey 1999-2018
Source: Front Immunol. 2024 Apr 4;15:1376544. doi: 10.3389/fimmu.2024.1376544 (PMC11024272; doi:10.3389/fimmu.2024.1376544)
Supplement: Supplementary file 1 [file DataSheet_1.docx]

**Systemic immune-Inflammation Index(SII) in sarcopenia and all-cause and cause-specific mortality in the United States**

[Figure S1.Kaplan-Meier curves show all-cause and cause-specific mortality differences by SII, excluding initial Cardiovascular diseases. 2](#_Toc88427334)

[Figure S2. Kaplan-Meier curves show all-cause and cause-specific mortality differences by SII, excluding Participants who died within two years. 3](#_Toc88427335)

[Figure S3. Kaplan-Meier curves show all-cause and cause-specific mortality differences by SII, excluding participants under the age of 45. 4](#_Toc88427336)

[Table S1. Association of SII with All-Cause and Cause-Specific Mortality in Sarcopenia Participants 5](#_Toc88427341)

[Table S2A. Subgroup of Association Between Systemic immune-Inflammation Index（SII） and Cardiovascular disease mortality 6](#_Toc88427342)

[Table S2B.Subgroup of Association Between Systemic immune-Inflammation Index（SII） and Cancer diseases mortality 6](#_Toc88427343)

[Table S2C.Subgroup of Association Between Systemic immune-Inflammation Index（SII） and Respiratory diseases mortality 6](#_Toc88427344)

[Table S3A.Characteristics of Sarcopenia: Participants Without Pre-existing CVD at Baseline in the NHANES Study 7](#_Toc88427345)

[Table S3B.Association of SII with All-Cause and Cause-Specific Mortality in Sarcopenia Participants Without Pre-existing CVD at Baseline 8](#_Toc88427345)

[Table S4A.Characteristics of Sarcopenia: Excluding Participants who Died within Two Years in the NHANES Study 9](#_Toc88427345)

[Table S4B.Association of SII with All-Cause and Cause-Specific Mortality in Sarcopenia Participants (Excluding Participants who Died within Two Years） 1](#_Toc88427345)0

[Table S5A.Characteristics of Sarcopenia Participants in the NHANES （Excluding Participants under the age of 45） 1](#_Toc88427345)1

[Table S5B.Association of SII with All-Cause and Cause-Specific Mortality in Sarcopenia Participants（Excluding Participants under the age of 45） 1](#_Toc88427345)2


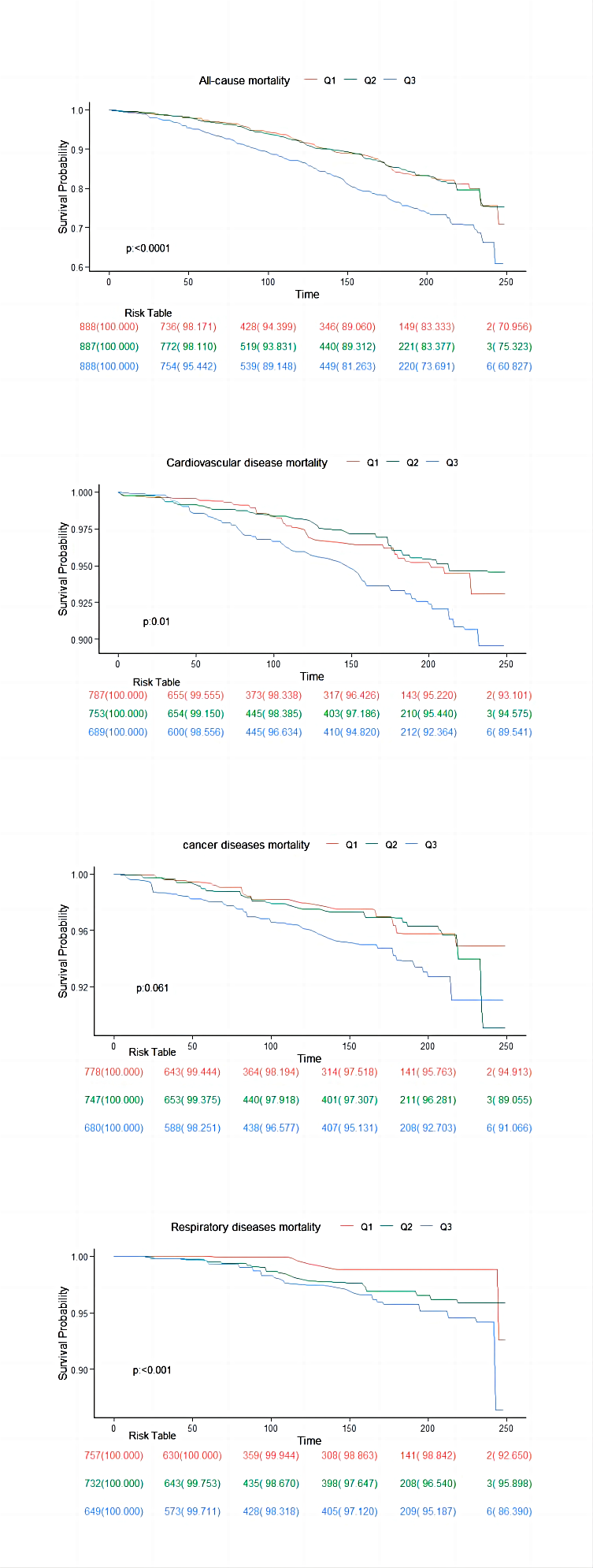


Figure S1.Kaplan-Meier curves show all-cause and cause-specific mortality differences by SII, excluding initial CVD.


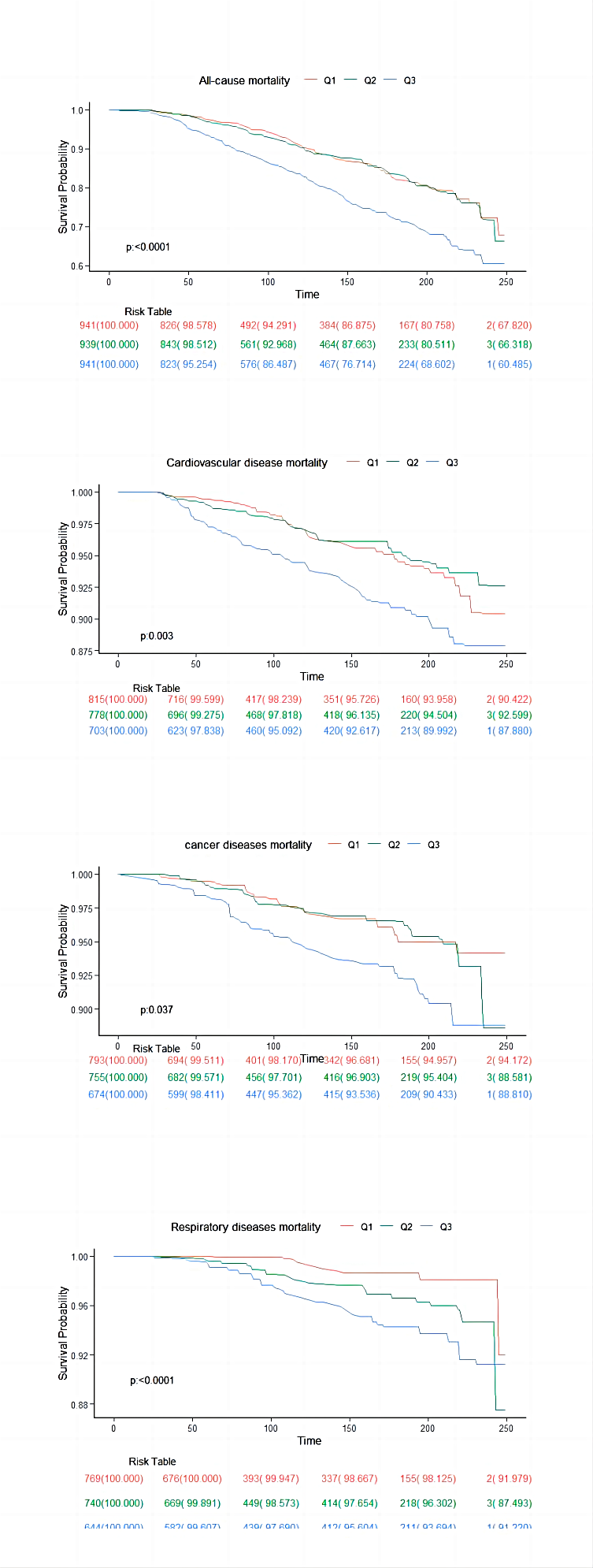


Figure S2. Kaplan-Meier curves show all-cause and cause-specific mortality differences by SII, excluding Participants who Died within Two Years.


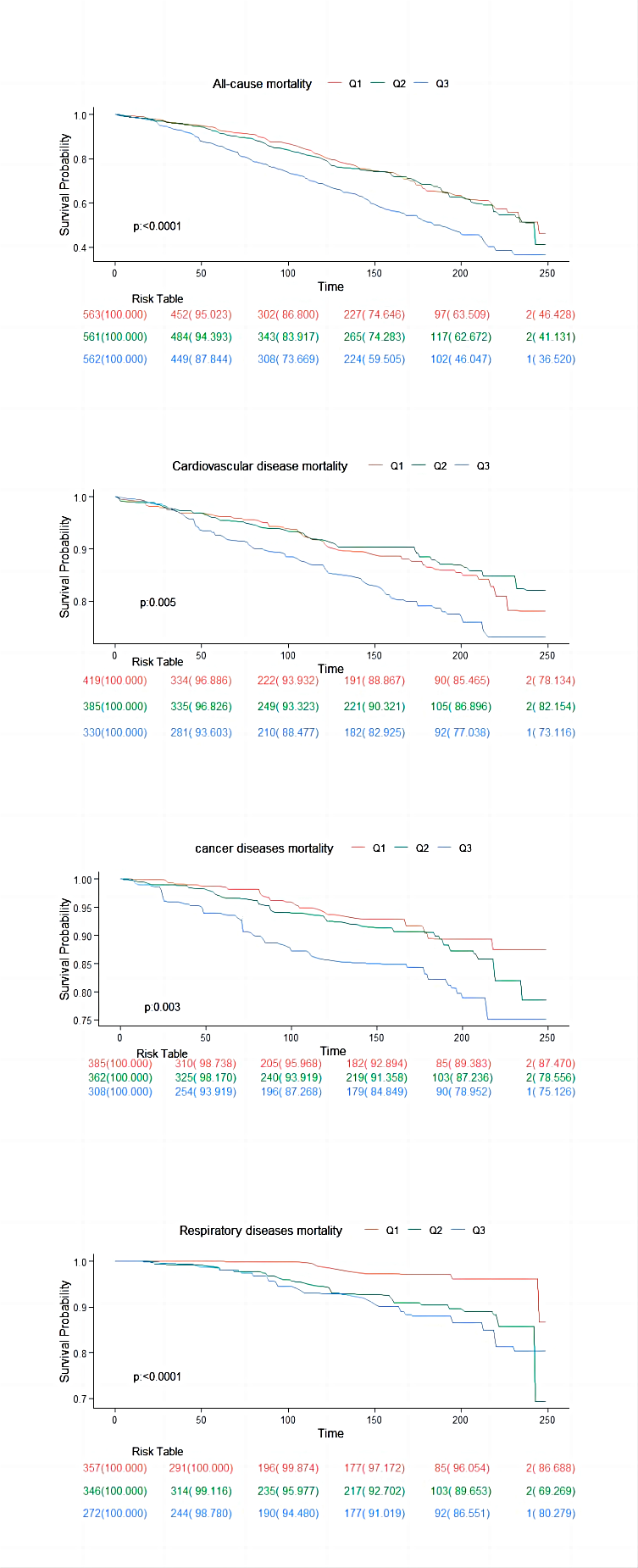


Figure S3. Kaplan-Meier curves show all-cause and cause-specific mortality differences by SII, excluding participants under the age of 45.


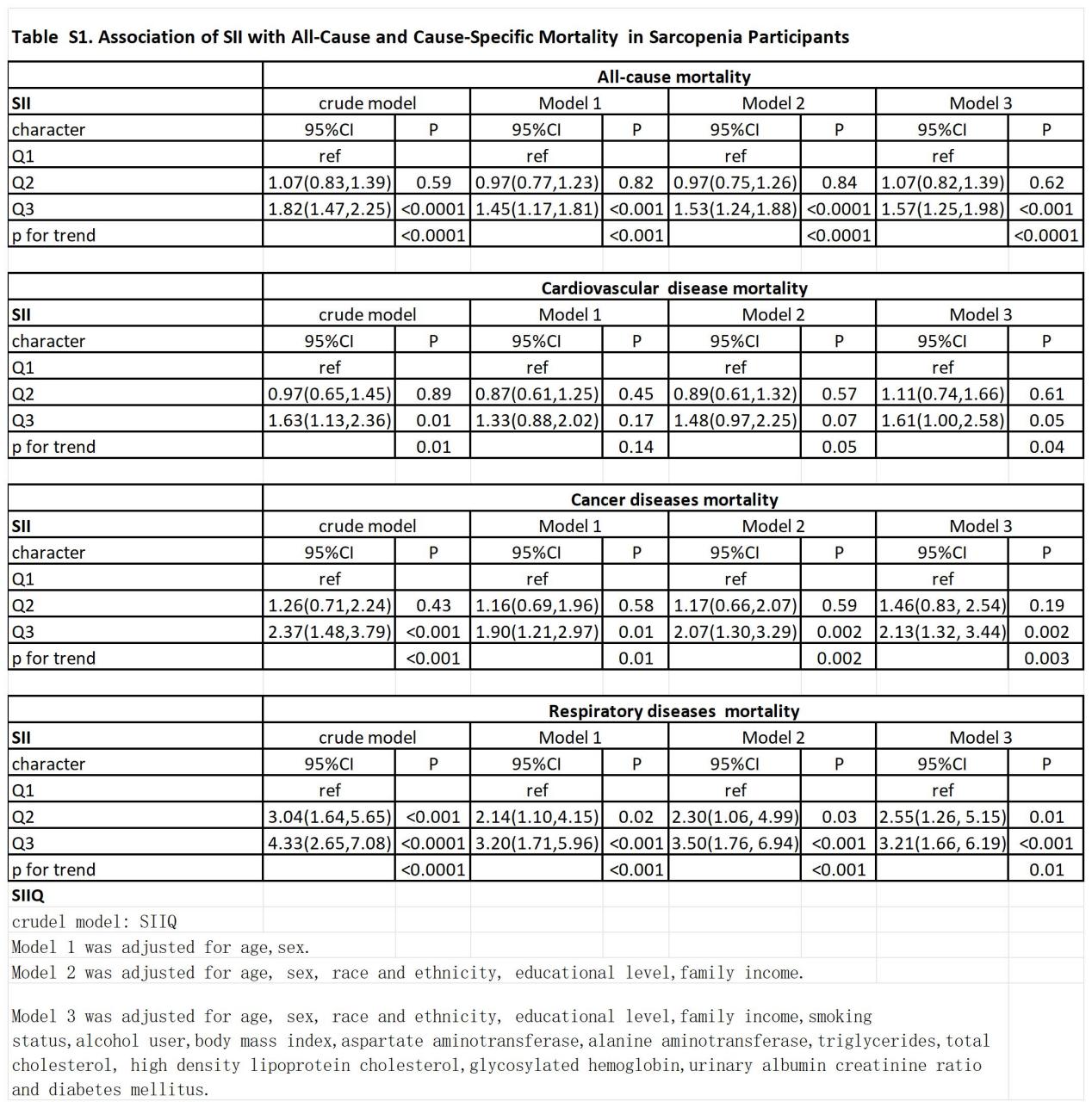


Table S1. Association of SII with All-Cause and Cause-Specific Mortality in Sarcopenia Participants .


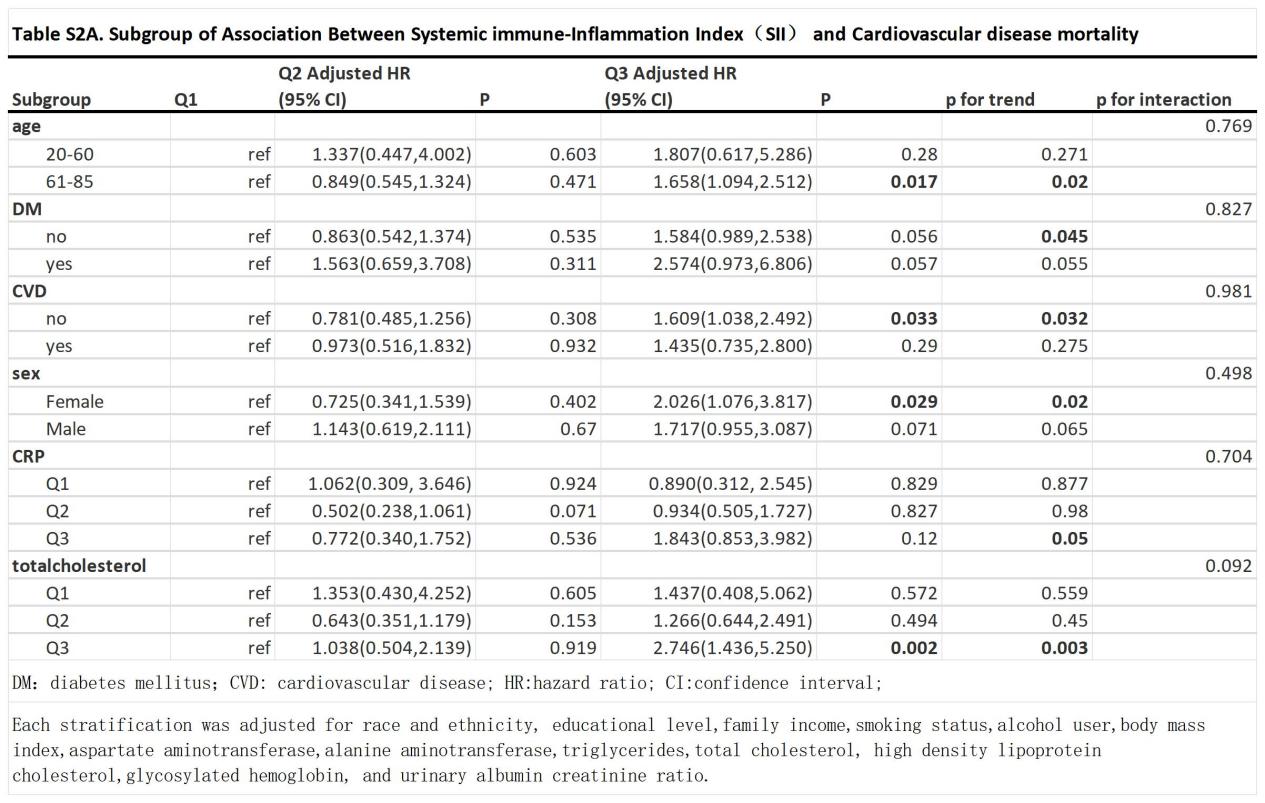


Table S2A. Subgroup of Association Between Systemic immune-Inflammation Index（SII） and Cardiovascular disease mortality.


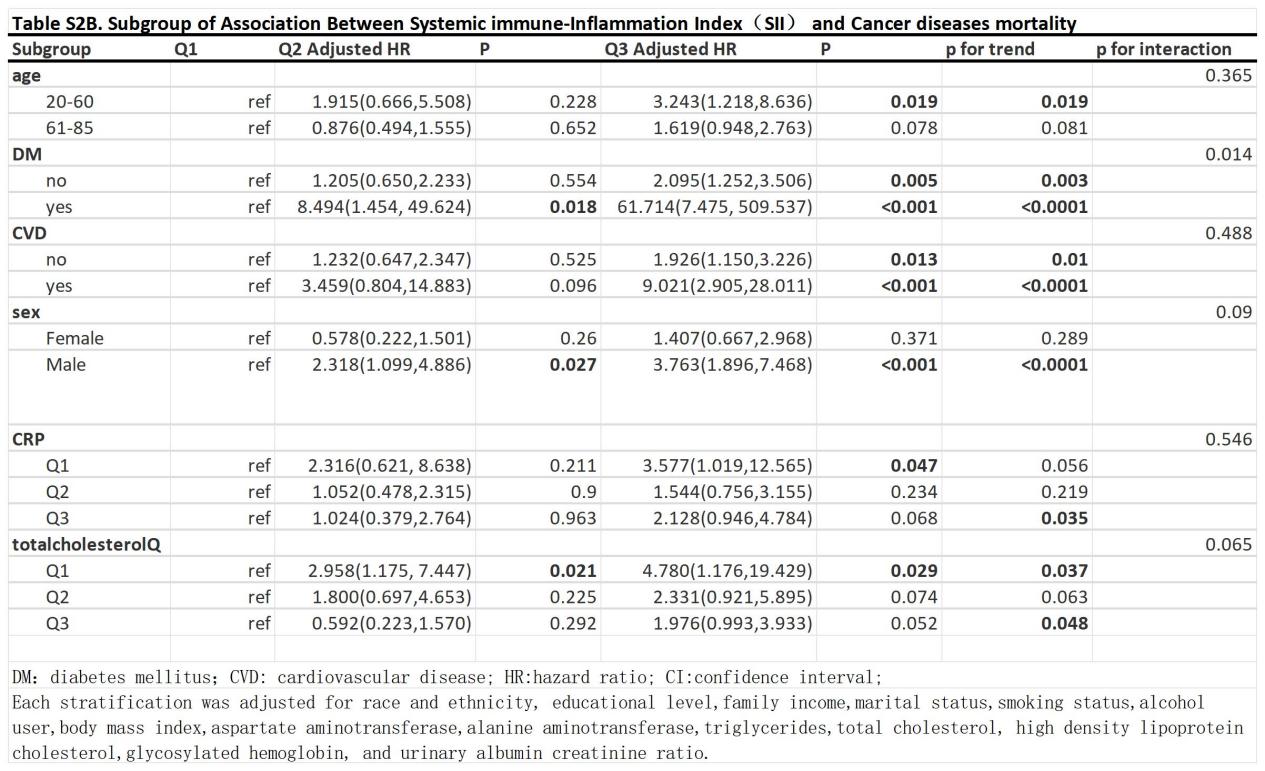


Table S2B.Subgroup of Association Between Systemic immune-Inflammation Index（SII） and Cancer diseases mortality.


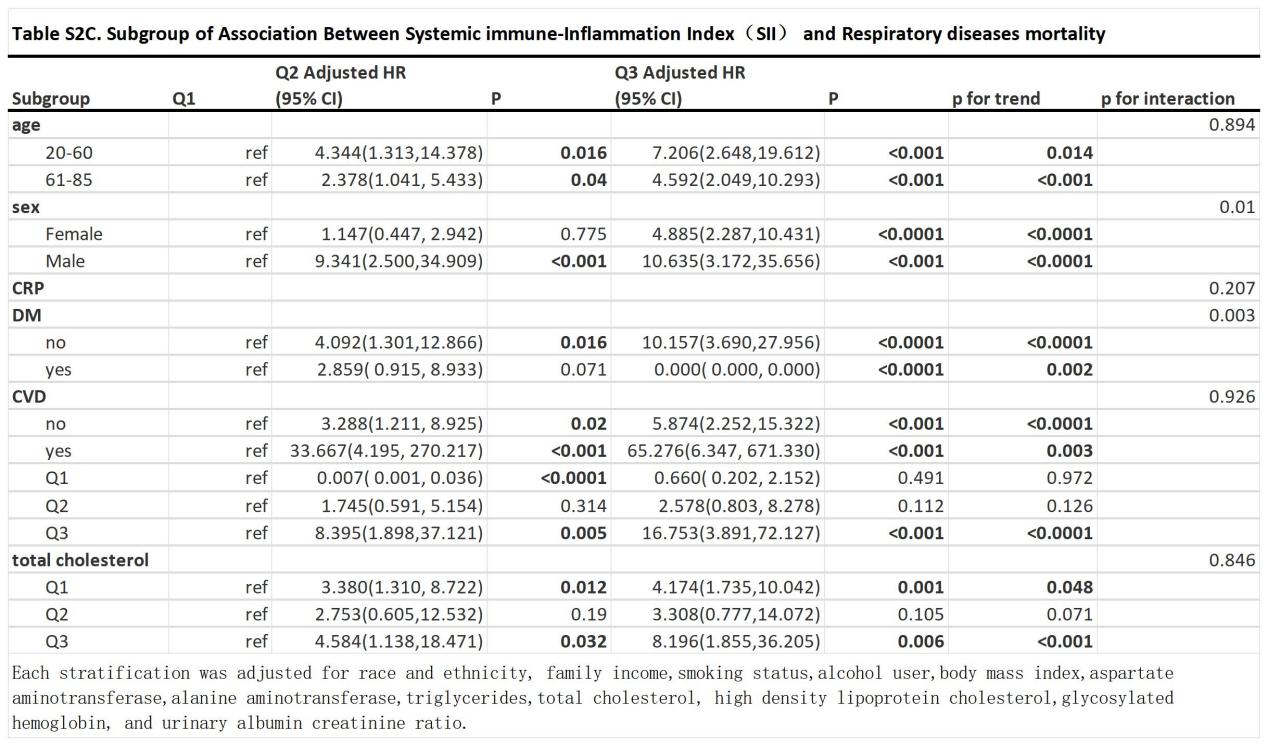


[Table S2C.Subgroup of Association Between Systemic immune-Inflammation Index（SII） and Respiratory diseases mortality](#_Toc88427344)


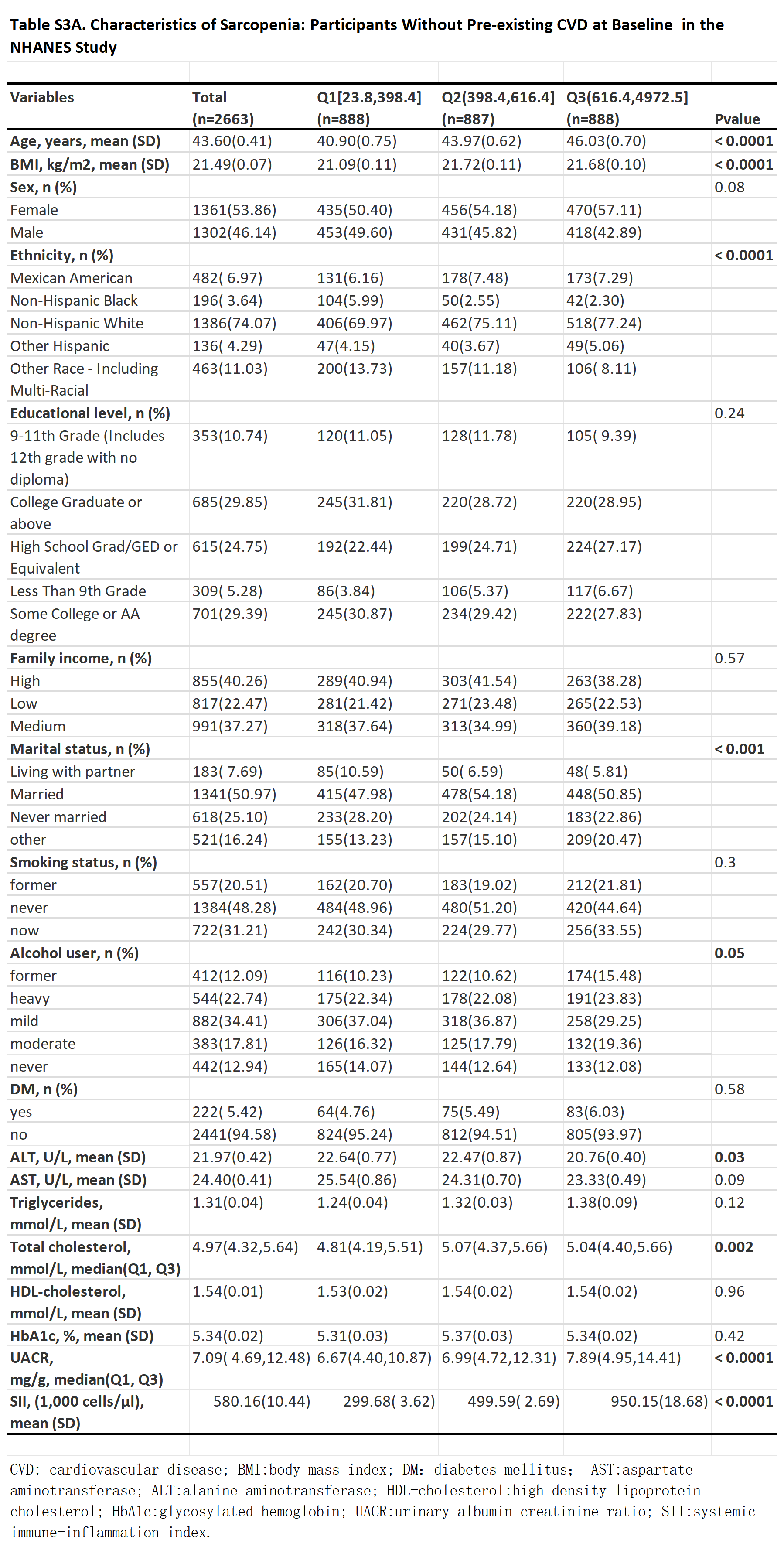


Table S3A.Characteristics of Sarcopenia: Participants Without Pre-existing CVD at Baseline in the NHANES Study


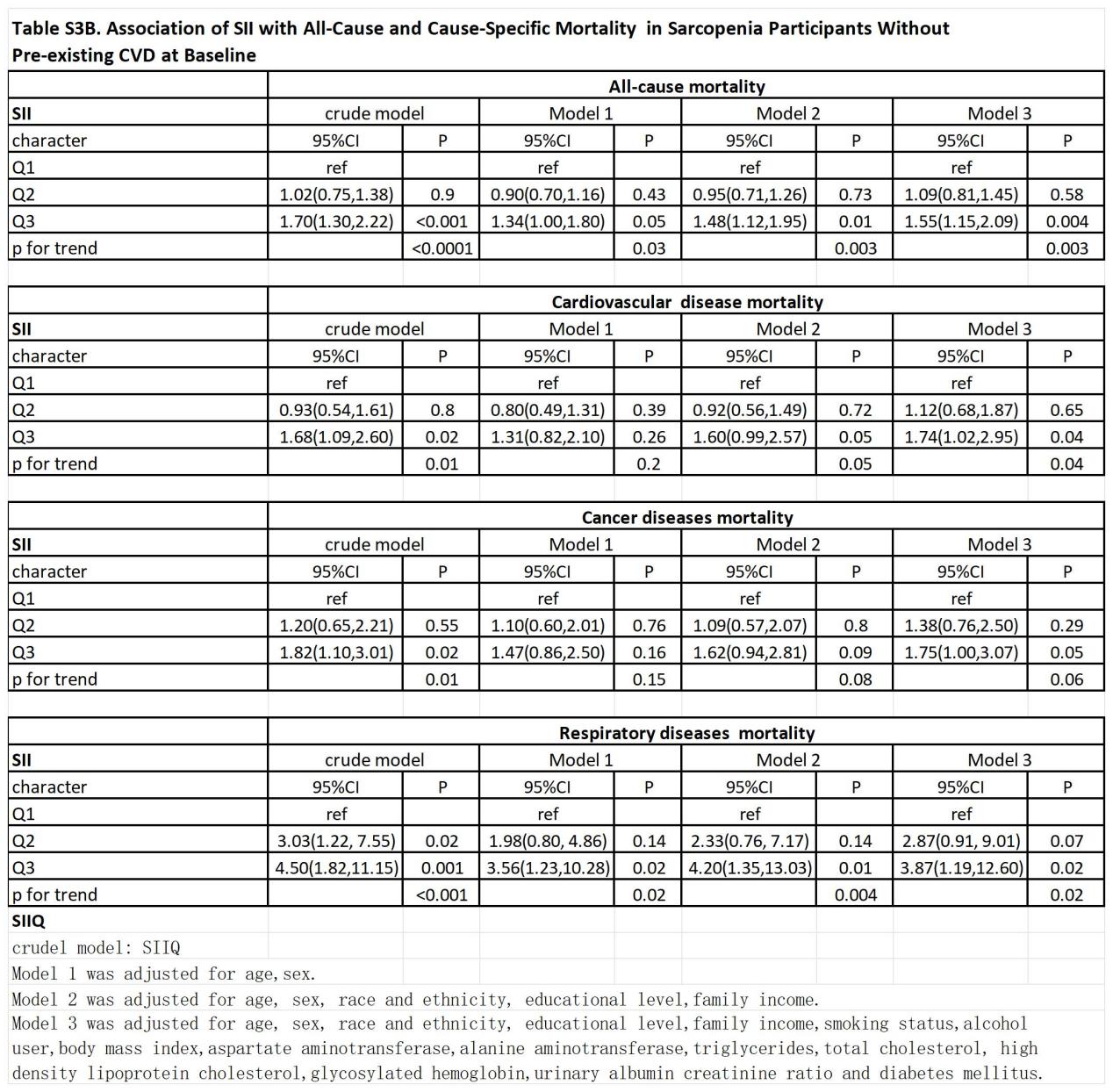


Table S3B.Association of SII with All-Cause and Cause-Specific Mortality in Sarcopenia Participants Without Pre-existing CVD at Baseline


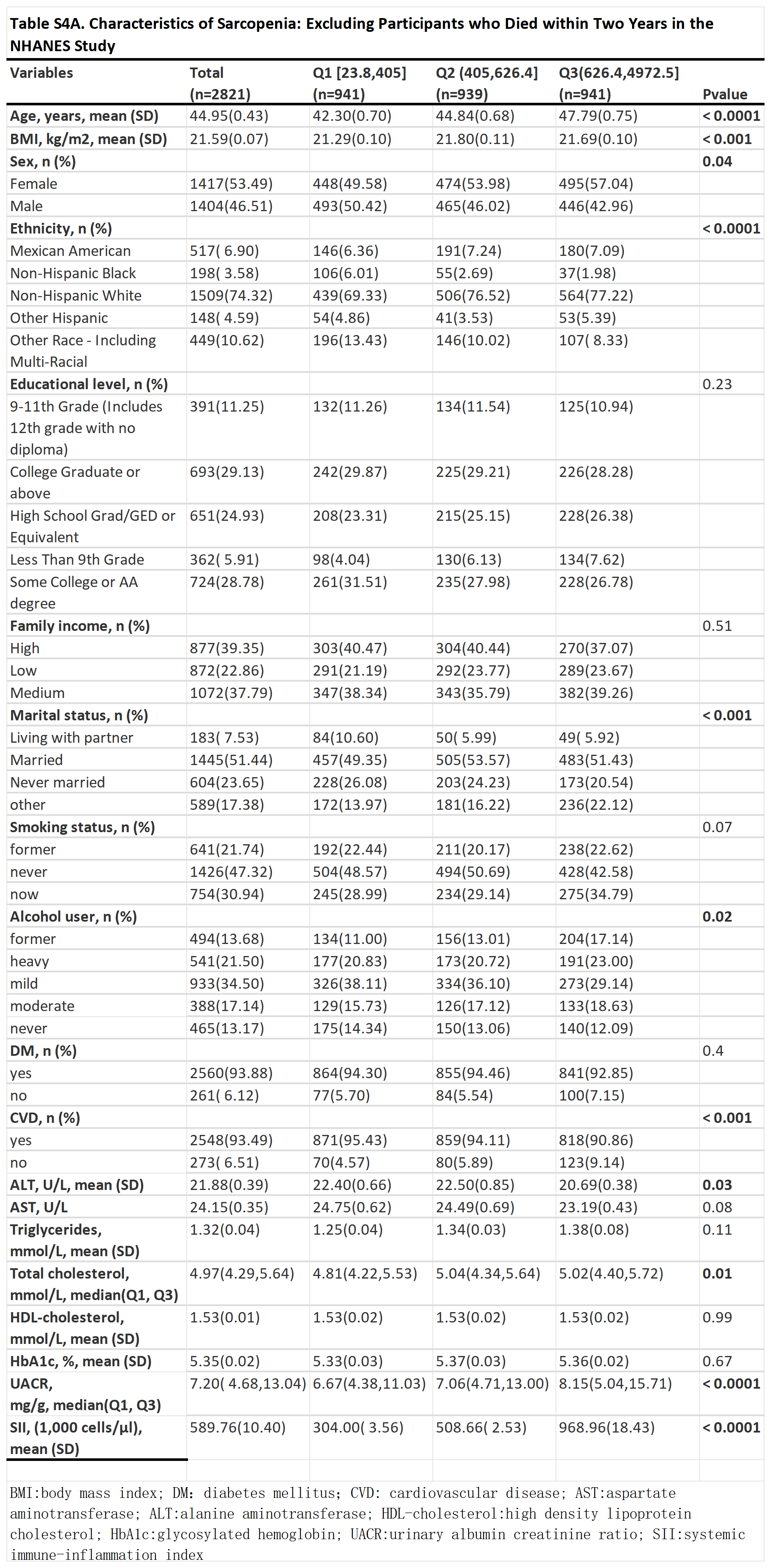


Table S4A.Characteristics of Sarcopenia: Excluding Participants who Died within Two Years in the NHANES Study


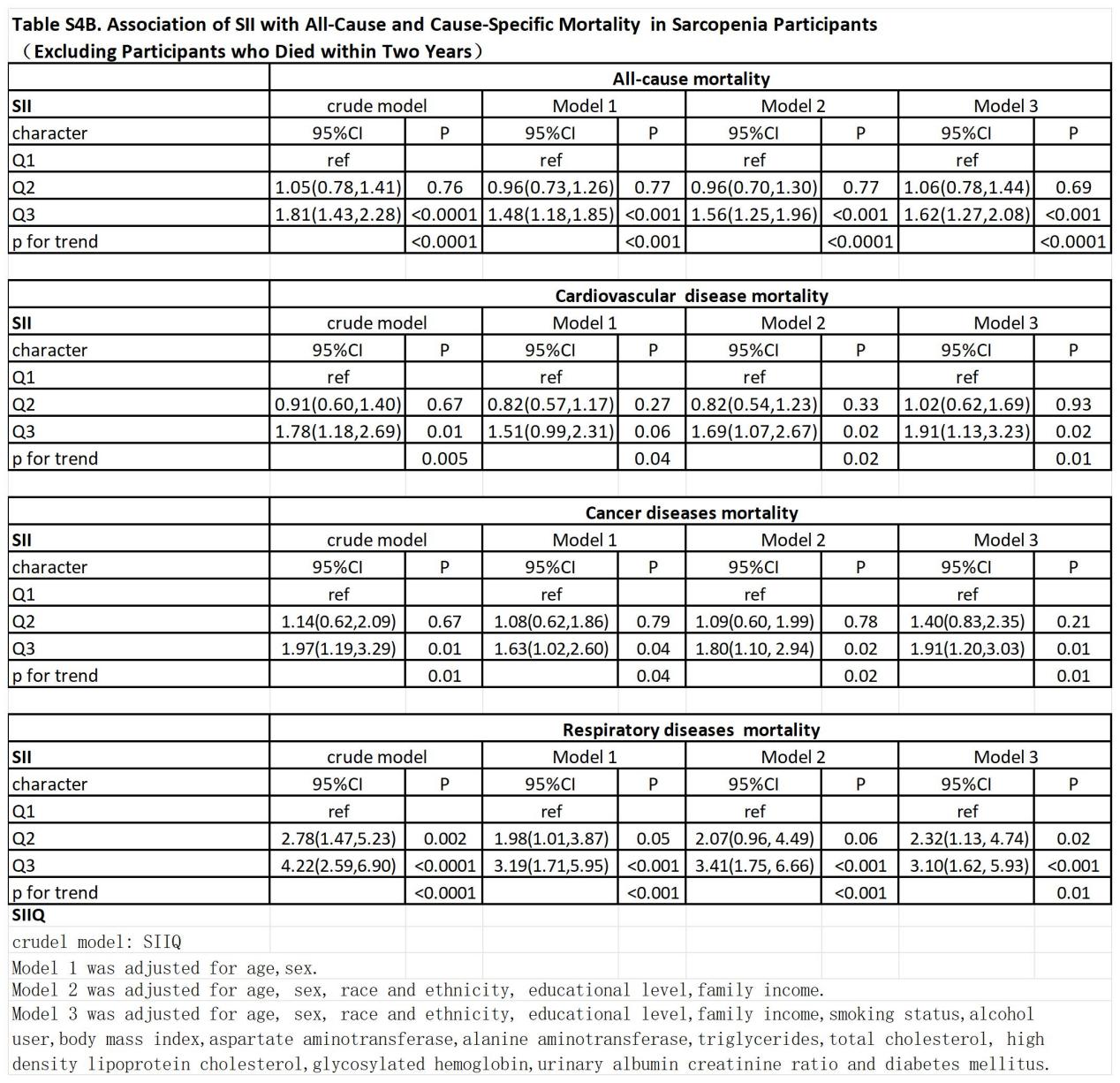


Table S4B.Association of SII with All-Cause and Cause-Specific Mortality in Sarcopenia Participants (Excluding Participants who Died within Two Years）


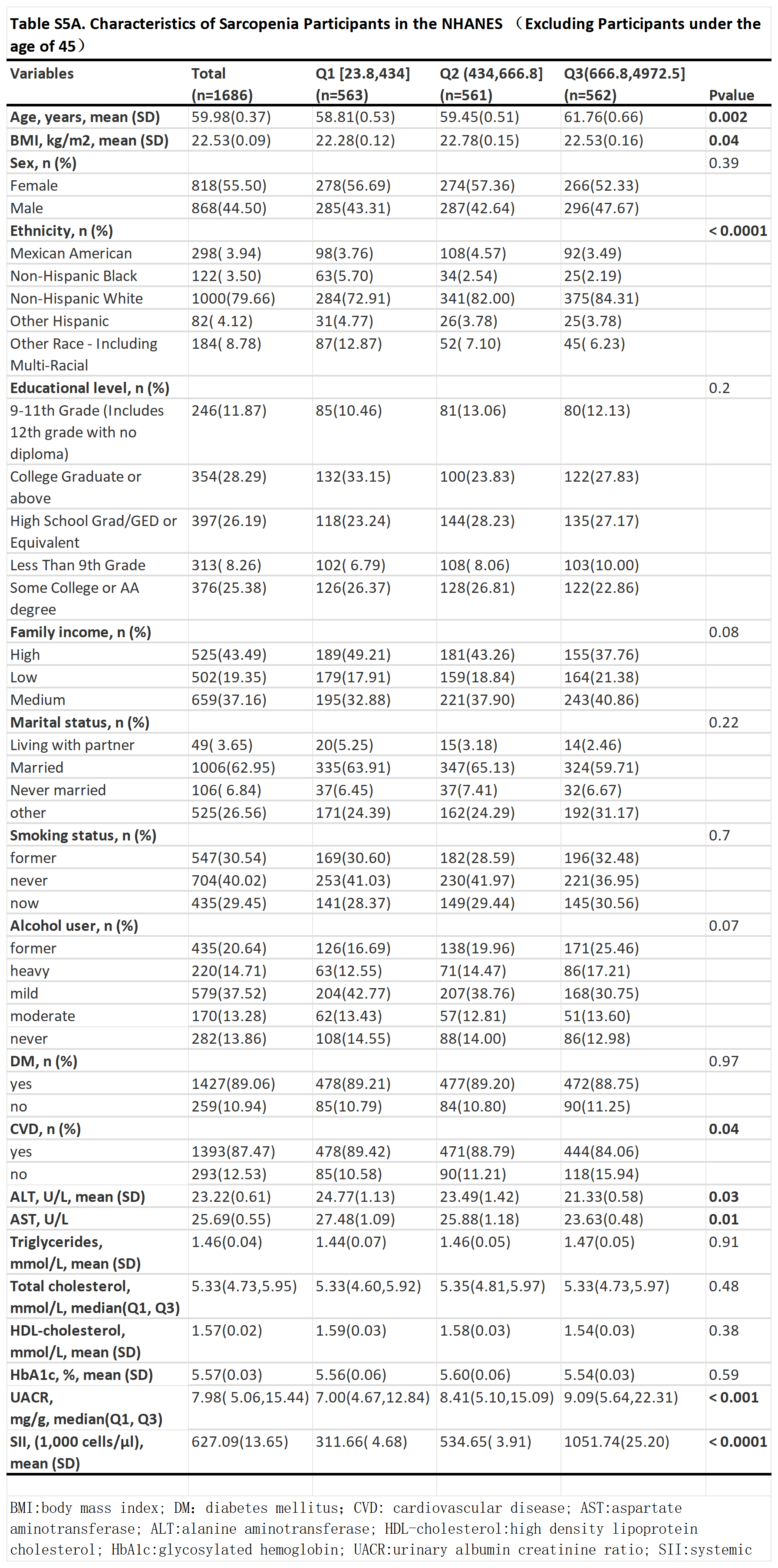


Table S5A.Characteristics of Sarcopenia Participants in the NHANES （Excluding Participants under the age of 45）


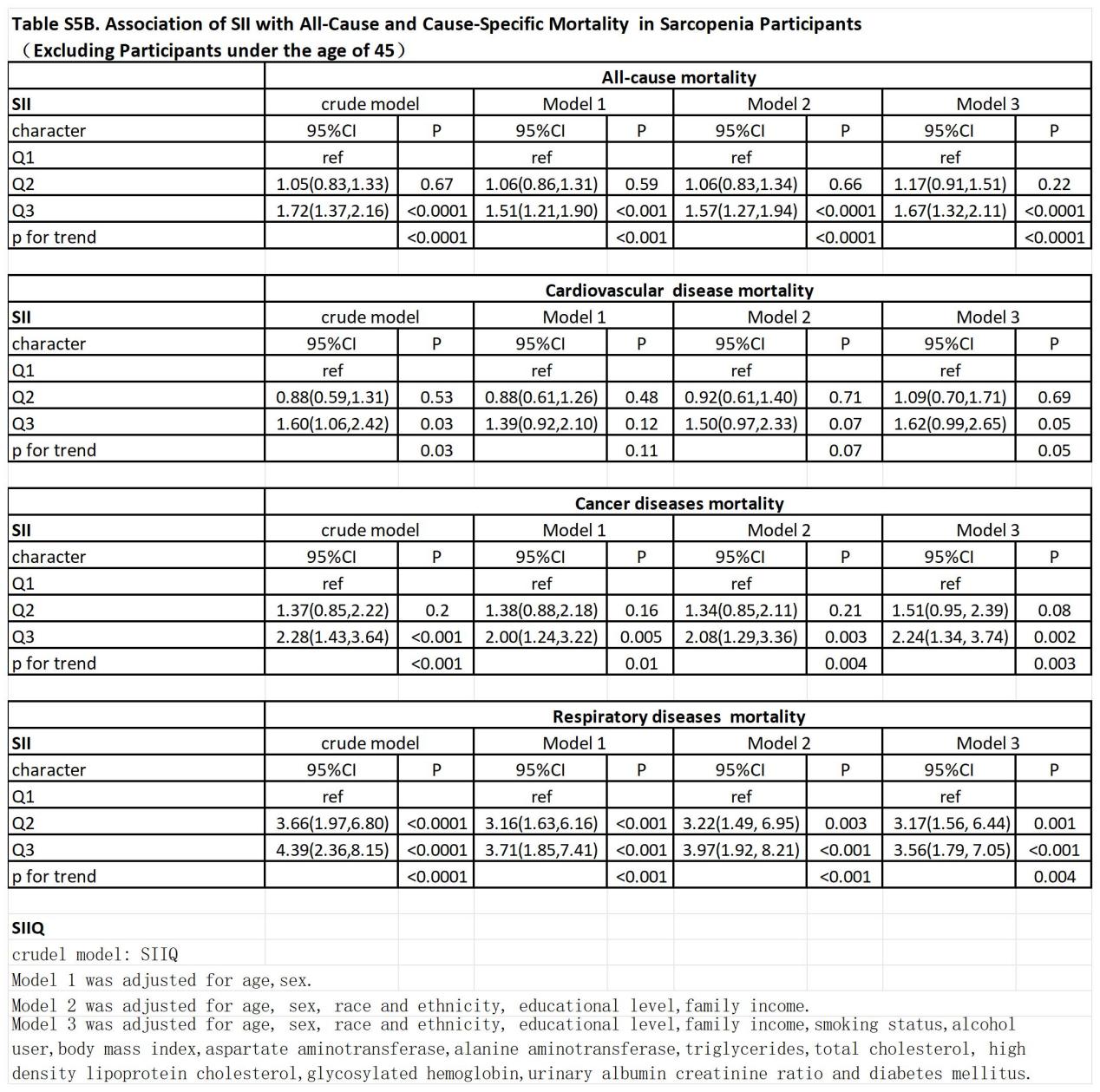


Table S5B.Association of SII with All-Cause and Cause-Specific Mortality in Sarcopenia Participants（Excluding Participants under the age of 45）
